# Diverse cultivation strategies are necessary to capture microbial diversity in High Arctic lake sediment

**DOI:** 10.3389/frmbi.2025.1619859

**Published:** 2025-09-26

**Authors:** Brittany M. Berdy, Claire E. Williams, Maria Sizova, Dawoon Jung, Nil Tandogan, Edgar D. Goluch, Slava Epstein

**Affiliations:** ^1^ Broad Institute of MIT and Harvard, Cambridge, MA, United States; ^2^ Department of Biology, Northeastern University, Boston, MA, United States; ^3^ Biology Department, University of Nevada, Reno, NV, United States; ^4^ Ningbo Institute of Marine Medicine, Peking University, Ningbo, Zhejiang, China; ^5^ Department of Chemical Engineering, Northeastern University, Boston, MA, United States

**Keywords:** microbial cultivation, *in situ* cultivation, uncultured microbiota, Arctic microbiology, microbial diversity

## Abstract

While metagenomics has revolutionized our understanding of microbial diversity and function, the cultivation of microorganisms remains indispensable for elucidating their physiological characteristics and potential biotechnological applications. Cultivation provides context to the vast metagenomic datasets and helps verify metagenome-based hypotheses on microbial interactions. The majority of microorganisms remain uncultivated, and this is particularly prominent from extreme environments such as the Arctic. Here we aimed to contribute to the growing body of work investigating microbial ecology in extreme environments by assessing the efficacy of a variety of cultivation approaches in lake sediment in the High Arctic. To try and capture the full breadth of organisms present, we used standard, *in situ*, and anoxic cultivation methods. We cultured a total of 1,109 microorganisms which clustered into 155 OTUs (97% rRNA gene sequence similarity), representing organisms from Proteobacteria, Actinobacteria, Bacteroidota, and Firmicutes. Importantly, no single method of cultivation proved to be sufficient to represent the cultivable organisms within the environment. Rather, each method resulted in many unique OTUs. Therefore, multiple approaches should be used in conjunction to access the bulk of microbial taxa in a given environment.

## Introduction

The advent of high-throughput sequencing technologies has revolutionized our understanding of microbial diversity, yet microbiology remains constrained by the inability to cultivate a significant proportion of microorganisms. Molecular approaches have estimated that the global diversity of microbiota ranges from 10^6^–10^9^ species, yet over 40 microbial phyla lack cultured representatives (Curtis et al., 2002; Lewis et al., 2021; Louca et al., 2019; Overmann et al., 2017). While culture-independent methods allow for the detection and analysis of uncultured organisms, cultivation of microbial isolates remains a necessary tool in microbiology.

Cultivation allows researchers to isolate microbial species to enable detailed study of their genetic makeup, metabolism, physiological, and biochemical properties, as well as for their use in bioprospecting. Cultivation is a key component of genetic studies to understand genetic function and is particularly critical given that many genes identified through sequencing lack functional annotations due to the lack of annotated genomes of cultured isolates (Laudadio et al., 2019). In addition, cultivation has enabled microbiologists to experimentally test hypotheses about microbial physiology and ecology, generate accurate taxonomic classifications, and study horizontal gene transfer events over time (Gill, 2017; Lewis et al., 2021; Poyet et al., 2019). Furthermore, cultivation is essential for understanding pathogenesis and remains a cornerstone of antibiotic susceptibility testing (Lagier et al., 2015). Through cultivation, scientists have unlocked groundbreaking advancements in biotechnology, medicine, and agriculture, including the discovery of CRISPR and PCR enzymes, antibiotics, and Bt pesticides (Brock and Freeze, 1969; Lewis, 2013; Mojica and Rodriguez-Valera, 2016; Yamamoto, 2001). Although molecular and computational approaches have facilitated the mining of genomic datasets to identify novel compounds, these methods are limited. Many compounds, including numerous antibiotics, are post-translationally modified by their host organisms—a process that cannot be replicated without access to the organism in culture (Lewis et al., 2010). Cultivation of previously uncultured microorganisms would have wide reaching and broad implications for medicine, ecology, and biotechnology.

Scientists have developed various strategies to improve the success of microbial cultivation. One common approach involves modifying growth media through supplementation, dilution, or targeted design based on genomic information, providing microbes with more optimal nutrients (Bashan et al., 2011; Bomar et al., 2011; Connon and Giovannoni, 2002; Greub, 2012). In order to isolate organisms from mixed populations, dilution to extinction has been commonly used (Button et al., 1993; Song et al., 2009). One promising cultivation approach is *in situ* cultivation, which leverages natural environmental nutrients to support microbial growth (Aoi et al., 2009; dos Santos et al., 2022; Jung et al., 2016; Kaeberlein et al., 2002; Liu et al., 2021; Nichols et al., 2010; Perrier et al., 2024). The general premise of *in situ* methods is that microbes are cultured in a growth chamber in their natural habitat. The use of membranes or other sub-micron openings allows for diffusion of growth factors and nutrients into the growth chamber, while restricting escape from the chamber by target organisms and isolating the microbes inside from surrounding competitors. These methods have been shown to enhance the richness, novelty, and diversity of cultured organisms (Bollmann et al., 2007; Gavrish et al., 2008; Jung et al., 2016, 2014; Kaeberlein et al., 2002).

In this study, we aimed to evaluate the effectiveness of a variety of *in situ* cultivation methods for capturing the breadth of the microbial diversity of a lake site in the High Arctic. We used an array of *in situ* cultivation approaches—diffusion chamber, trap, filter plate, Itip, and a microfluidic device (iPore)—and compared these to standard cultivation in a petri dish (Bollmann et al., 2007; Gavrish et al., 2008; Jung et al., 2014; Kaeberlein et al., 2002; Tandogan et al., 2014). We chose to survey the uppermost layer of sediment of a lake site outside of Thule, Greenland (76°32.659’ N, 68°27.458’ W). Unlike temperate environments, which have been known to host DNA from up to 50,000 distinct species (Roesch et al., 2007), this Arctic site provided a more tractable system for our investigation. Additionally, we incubated a subset of diffusion chambers and standard plates under anoxic conditions to assess whether anoxic incubation enhanced microbial recovery in a sediment layer with unknown oxygen content. Our results indicated that no single cultivation method was sufficient to represent the full spectrum of organisms in the environment. Instead, a combination of methods was required to maximize the diversity, richness, and novelty of microbial species isolated. These findings highlight the importance of integrating multiple cultivation approaches to comprehensively study microbial communities in complex environments.

## Materials and methods

To cultivate biologically active and relevant microorganisms within our study community we employed standard and *in situ* cultivation methodologies. All *in situ* devices were constructed in-house.

### *In situ* devices

#### Diffusion chamber

The diffusion chamber was constructed using a stainless-steel O-ring with 0.03 µm polycarbonate membrane affixed to either side using silicone glue to create a growth chamber as previously described (Kaeberlein et al., 2002). The membrane allows exchange of chemicals and growth factors between the environment and the growth chamber while restricting cell movement. A sediment–agar mix was placed in the chamber (details below) and following solidification of the agar, the top of the device was sealed with another 0.03-µm pore-size membrane using silicone glue. Sealed chambers were incubated *in situ* just below the sediment surface (~3 mm).

#### Trap

To enrich for filamentous, chain forming, and motile organisms, we constructed a microbial trap. The trap was constructed in the same manner as the diffusion chamber, except traps were filled with sterile 1% agar. One side of the trap was sealed with a 0.3-µm polycarbonate membrane, while the other side was sealed with a 0.4-µm polycarbonate membrane to allow for microbial colonization of the device as described in (Gavrish et al., 2008). Traps were placed on the surface of the sediment with the 0.4 µm pore side face down for microbial entry.

#### Filter plate microbial trap

The FPMT is a high-throughput adaptation of the trap described above, featuring 96 individual small chambers that prevent fast-growing bacteria from spreading between compartments. FPMT plates were constructed as described in (Jung and Ahn, 2012). In brief, FPMT plates contained 96 wells, each serving as a small growth chamber. The bottom of each well was fitted with a hydrophilic polyvinyldenefluoride (PVDF) membrane with 0.45µm pore large enough to allow for microbial entry. Wells were filled with sterile 0.7% agar and the device was placed on top of the sediment to allow direct contact of the membrane with the target environment for microbial colonization.

#### Itip

Itips were constructed as described in (Jung et al., 2014). Briefly, the lower portion of a sterile 200 µL pipette tip was filled with acid-washed glass beads of various sizes (60–200 µm in diameter) to prevent the invasion of larger organisms. Sterilized media, as described below, was mixed with 0.7% agar and added above the glass beads. The narrow tip of the Itips were placed just under the surface of the sediment. Organisms were able to enter the device through the narrow top opening, while the opposite end was sealed with waterproof silicone adhesive.

#### iPore

The theory, design, and proof of concept for the microfluidic devices (iPores) used for microbial isolation was published previously (Tandogan et al., 2014). iPore devices consist of a small entry pore leading to long constriction channels terminating in growth chambers. The main premise of the iPore design is to utilize microbe-sized constrictions to prevent multiple species from colonizing the same growth chamber. The iPore is placed in the environment, and microbes can enter through the main entrance and move toward narrower constrictions leading to an isolation chamber. The constrictions and chambers were filled with DI water and a 0.03 um membrane sealed the outside of the growth chamber allowing for diffusion of nutrients from the environment into the growth chambers. The constrictions are designed to be narrow enough so that the cross-sectional area should only permit one single cell to enter—thus blocking the opening from additional cells. As the entering cell grows and divides through the constriction, it will propagate within the isolation chamber. A variety of constriction channel widths and lengths were used to try and capture an array of species from the sediment.

### Sample site

​​Sediment samples were collected from the upper (oxic) layer of an artificial lake in Northwest Greenland, outside of Thule Airbase (N 76°32.659’ W 68°27.458’). This lake was chosen based on a preliminary survey of multiple locations around Thule Airbase in 2013. For this study, two sample sites within the lake were chosen, designated as Rich Lake 1 (RL1) and Rich Lake 2 (RL2). Sediment from each site was combined for cultivation. Markers (plastic pipes dug into the ground) were placed at both sites to ensure continuous sampling from the same location throughout the season. The sites were 30 feet from each other, about 15 cm from the water edge, with 1–3 mm of water above the sediment. Sediment samples were collected at various time points during the summer of 2014 for cultivation (**Table 1**). The temperature and pH of the sediment was measured periodically and stayed essentially unchanged throughout the season: 10 °C with a pH of 6.8.

**Table 1:** Sampling dates and incubation durations for standard and *in situ* cultivation devices.

Method	Date of sample collection		
Standard cultivation		Sample collection	
Standard cultivation 1		11-Jun	
Standard cultivation 2		08-Jul	
Standard cultivation 3		22-Jul	
*In situ* cultivation	Date of device set up	Device retrieval	Incubation time (days)
*In situ* 1			
Diffusion chamber 1	05-Jun	26-Jun	22
Trap 1	05-Jun	26-Jun	22
Filter plate 1	05-Jun	27-Jun	23
Itip 1	05-Jun	27-Jun	23
*In situ* 2			
Diffusion chamber 2	30-Jun	15-Jul	16
Trap 2	30-Jun	15-Jul	16
iPore	03-Jul	16-Jul	13
*In situ* 3			
Diffusion chamber 3	29-Jul	08-Aug	11
Trap 3	29-Jul	08-Aug	11
Filter plate 3	29-Jul	08-Aug	11
Itip 3	29-Jul	08-Aug	11
iPore	24-Jul	06-Aug	13

### Cultivation conditions

Three cultivation media were used: R2A, 1:100 Nutrient Agar (1:100 NA) and Soil Extract Agar (SE). R2A was made following the manufacturer recommendations (BD, Difco 218263). A 1:100 dilution of Nutrient Agar was made using 0.8 g/L Nutrient Broth (Difco 247940) and Bacto Technical Agar (15 g/L; Difco 281230). Sediment from the lake was mixed with DI water and sterilized at 121°C and 15 PSI for one hour. The solution was allowed to sediment and the supernatant was collected. For SE agar, Bacto Technical Agar (15 g/L) was added to the sediment and autoclaved. The average temperature of the lake throughout the entire sampling campaign was 10°C, however the sediment experiences colder temperatures during other parts of the season (such as 2°C). To simulate the natural conditions of the lake, all cultures were incubated at both 0–2°C and 10°C.

### Standard cultivation

Samples for standard cultivation were collected at three time points between June and July 2014 (**Table 1**) from both RL1 and RL2. Using a sterile teaspoon, the uppermost 2–3 mm of sediment was transferred to a 50 mL Falcon tube and immediately transported to the laboratory within an hour. RL1 and RL2 samples were combined and vortexed. Serial dilutions through 10^–5^ were prepared using phosphate-buffered saline. Each dilution was plated on three different media: R2A, a 1:100 dilution of NA, and SE. To capture facultative anaerobes, a subset of R2A and NA plates were concurrently incubated in anaerobic boxes under 95% nitrogen and 5% carbon dioxide at room temperature.

### *In situ* cultivation

*In situ* devices were constructed as described above. Sediment for diffusion chamber inoculum was collected and serial dilutions were prepared in 10mL of 42°C warm agar, and 3 mL was loaded into each diffusion chamber, which was then sealed with a polycarbonate membrane. Traps and FPMTs were filled with sterile agar. Itips were filled with either R2A, a 1:100 dilution of NA, or SE. *In situ* devices were placed at the sample site 3 times throughout the season (**Table 1**) and left to incubate for 2–3 weeks (**Supplementary Figures S1, S2**).

### Device and biomass retrieval

After incubation, all devices (9 DCs, 6 Traps, 1 filter plate, 9 Itips per site) were aseptically disassembled with a sterile blade and agar containing microorganisms was carefully removed using sterile loops. The agar mixture was combined with sterile media, homogenized, and vortexed. The mixture was used as an inoculum for serial dilutions through 10^−5^, of which 100 µl of each dilution were spread on three types of solid media: R2A, a 1:100 dilution of nutrient agar, and soil extract agar, and incubated at 0°C or 10°C. Contents of the growth chamber in iPore devices were retrieved with a sterile toothpick and streaked directly on agar plates.

### Microbial isolation and sub-cultivation

Standard cultivation plates and plates from serial dilutions of *in situ* devices were incubated at 0°C or 10°C for at least three weeks. Following incubation, plates were individually examined and dilutions resulting in single colonies were selected. Biomass from single colonies was lifted off the plate with a toothpick, restreaked, and incubated on the same media and at the same temperature as the parent plate. To the best of our ability, colonies were picked to encompass as many different representative phenotypes as were distinguishable under a dissecting scope. Denser and less dense plates were also examined under the dissecting scope for additional unique phenotypes, which were isolated as described above.

### Isolation, identification, and downstream analysis

Sealed petri dishes were transported in an enclosed container with ice packs to Northeastern University, Boston MA, U.S.A. and immediately returned to their original cultivation temperature. Isolates were cultured on either 1% NA, 10% NA, or R2A until determined pure by microscopic visualization. Pure isolates were archived in 20% glycerol at −80°C. Taxonomic identification was performed by sequencing the 16S rRNA gene. Biomass from a colony was picked with a sterile toothpick and homogenized with molecular-grade water for colony PCR. One microliter of homogenate was used as a template for PCR-enabled 16S Sanger Sequencing using the 27F (5’-AGAGTTTGATCCTGGCTCAG-3’) and 1492R (5’-GGTTACCTTGTTAGGACTT-3’) primers (Lane, 1991) and the HotStarTaq system (Qiagen Cat #203445). PCR was performed under the following conditions: 15-minute denaturation at 95°C, followed by 20 cycles of 1 minute at 95°C, 1 minute at 55°C, and 1 minute at 72°C. PCR products were purified and sequenced commercially (at Macrogen or Genewiz) by fluorescent terminator sequencing using the 27F primer. Some isolates were re-sequenced in the case of poor quality with the use of the 1492R primer.

In total 1109 isolates were sequenced (**Table 2**). The sequences were assessed for quality and manually trimmed. After trimming low-quality bases, the average sequence length was 772 base pairs (standard deviation=129 bp). Sequences were imported into QIIME2 version 2022.8 (Bolyen et al., 2019) and dereplicated using VSEARCH (Rognes et al., 2016). Sequences were clustered at 97% sequence similarity into OTUs, and taxonomy was assigned using full length 16S reference sequences from the SILVA taxonomy database release 138 (Quast et al., 2013). A phylogenetic tree was generated and rooted using the Mafft and fasttree QIIME2 plug-ins (Katoh et al., 2002; Price et al., 2010). Figures were constructed using *phyloseq* (McMurdie and Holmes, 2013), *ggplot2* (Hadley Wickham, 2016) and *ggtree* (Yu, 2020; Yu et al., 2017) in R (version 4.2.3) (R Core Team, 2021).

**Table 2:** Number of colonies isolated, categorized by cultivation method and subculture media type.

Method of isolation	R2a	SE	NA	Total
Standard cultivation	161	67	91	319
Standard cultivation (anoxic)	20	0	0	20
Diffusion chamber	186	55	104	345
Diffusion chamber (anoxic)	44	2	6	52
Trap	37	16	29	82
Itip	10	8	60	78
Filter plate	107	3	0	110
iPore	103	0	0	103
Total	668	151	290	1109

To compare cultivation approaches to each other, isolation power was calculated as the number of OTUs captured by a method, divided by the total number of OTUs observed across the study. Isolation efficiency was calculated by dividing the isolation power by the number of isolates from that method.

## Results

### Overview of cultivated organisms

We isolated a total of 1109 colonies and sequenced these isolates using Sanger sequencing of the 16S rRNA gene (**Table 1**). After sequencing, quality control, and removal of non-bacterial sequences we retained 1,093 sequences. These sequences clustered into 155 97% rRNA gene sequence similarity based OTUs. OTUs were classified within four bacterial phyla: Proteobacteria, Actinobacteria, Bacteroidota, and Firmicutes (nomenclature based on SILVA release 138). Proteobacteria was the most abundant phylum in our isolate collection, encompassing 58.6% of cultured isolates. Within the Proteobacteria phylum, we cultivated representatives of Alpha- and Gammaproteobacteria. Our sequenced isolates spanned 77 genera, but two genera dominated, with 19.1% of isolates classified as *Flavobacterium* and 10.5% as *Pseudomonas*. We also cultivated a high level of intra-generic diversity of *Flavobacterium*, isolating 19 unique *Flavobacterium* OTUs.

### *In situ* and standard cultivation produce diverse and unique culture collections

We used an array of cultivation approaches—standard (aerobic and anoxic), and *in situ*—to establish a representative culture collection of the lake sediment. Using aerobic standard cultivation, we cultured 319 isolates which clustered into 92 OTUs. Using anoxic standard cultivation, we cultured 20 isolates and 5 OTUs. Among all *in situ* approaches, we cultured 770 isolates and 104 OTUs.

Standard, anoxic, and *in situ* cultivation techniques yielded diverse collections of isolates. Both standard and *in situ* approaches resulted in cultured isolates spanning 4 different phyla, while anoxic approaches resulted in cultured isolates from 2 phyla. *In situ* approaches resulted in isolates spanning 59 unique genera across 28 families and 18 orders. Standard approaches resulted in isolates from 48 unique genera across 27 families and 18 orders. Anoxic cultivation resulted in isolates from 4 genera, 3 families, and 2 orders.

**Figure 1:**
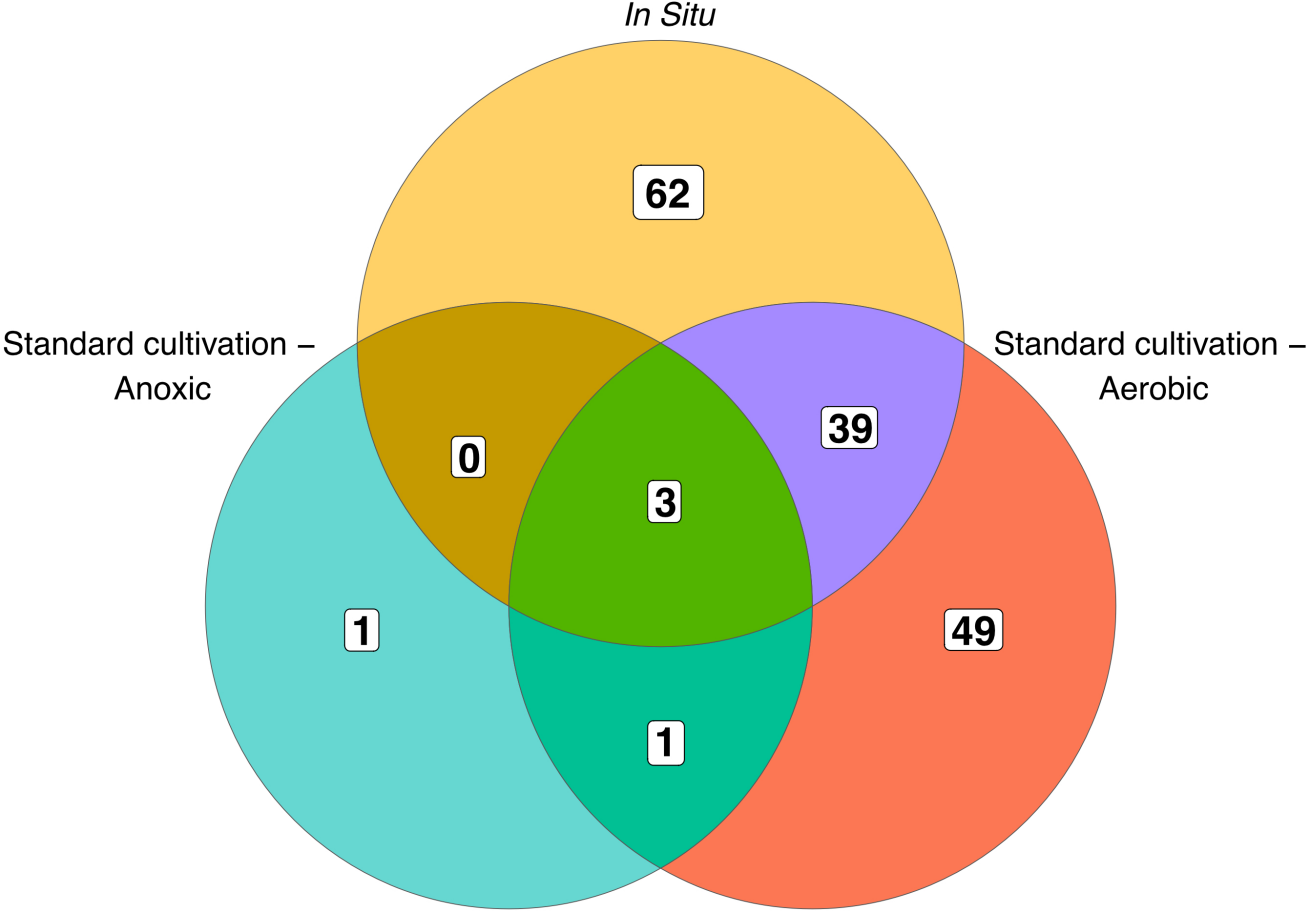
Overlap of OTUs cultured by each approach. Numbers within the diagram indicate OTUs unique to, or shared between, the different cultivation methods. Standard aerobic and anoxic cultivation methods are shown separately, while all *in situ* approaches are combined into a single category.

**Figure 2:**
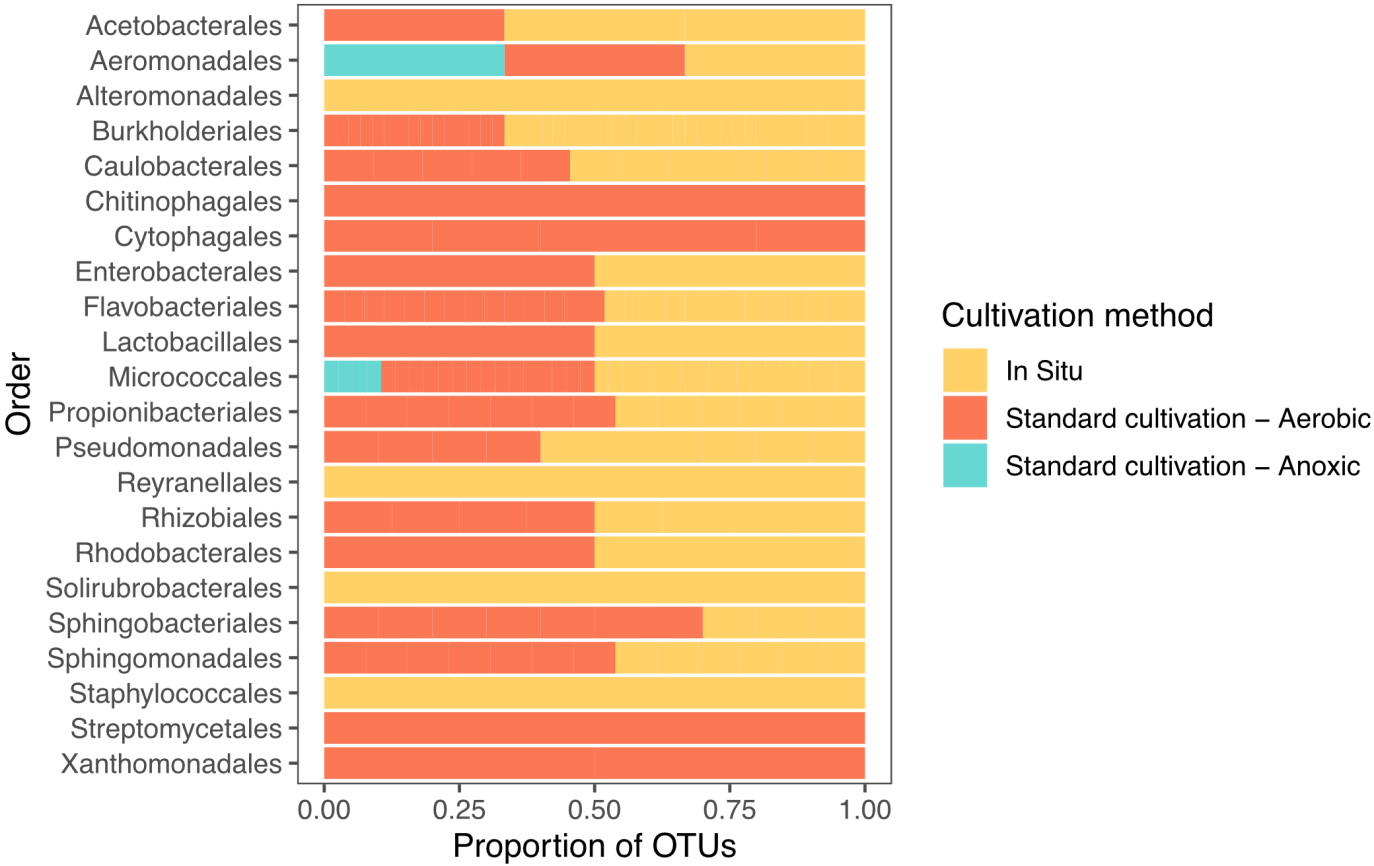
Distribution of recovered isolates, grouped by the taxonomic order of their corresponding OTUs, across different cultivation methods. Bars represent the proportion of isolates associated with each cultivation strategy (*in situ*, standard aerobic, and standard anoxic cultivation).

When comparing the OTU level composition of our isolate collections, we observed minimal overlap of isolates obtained by the different approaches: only 3 of 155 unique OTUs were common among all three broad methodologies (**Figure 1**). Using *in situ* cultivation methods, we successfully cultured 62 OTUs that were not recovered through standard cultivation alone. Conversely, traditional methods yielded 49 OTUs that *in situ* approaches failed to capture. These differences were also apparent when comparing which orders were successfully cultivated by these 3 broad approaches (**Figure 2**). In particular, there were 4 bacterial orders for which isolates were only cultivated using *in situ* approaches, and 4 that were only captured using standard cultivation.

### Individual *in situ* approaches produce unique culture collections

**Figure 3:**
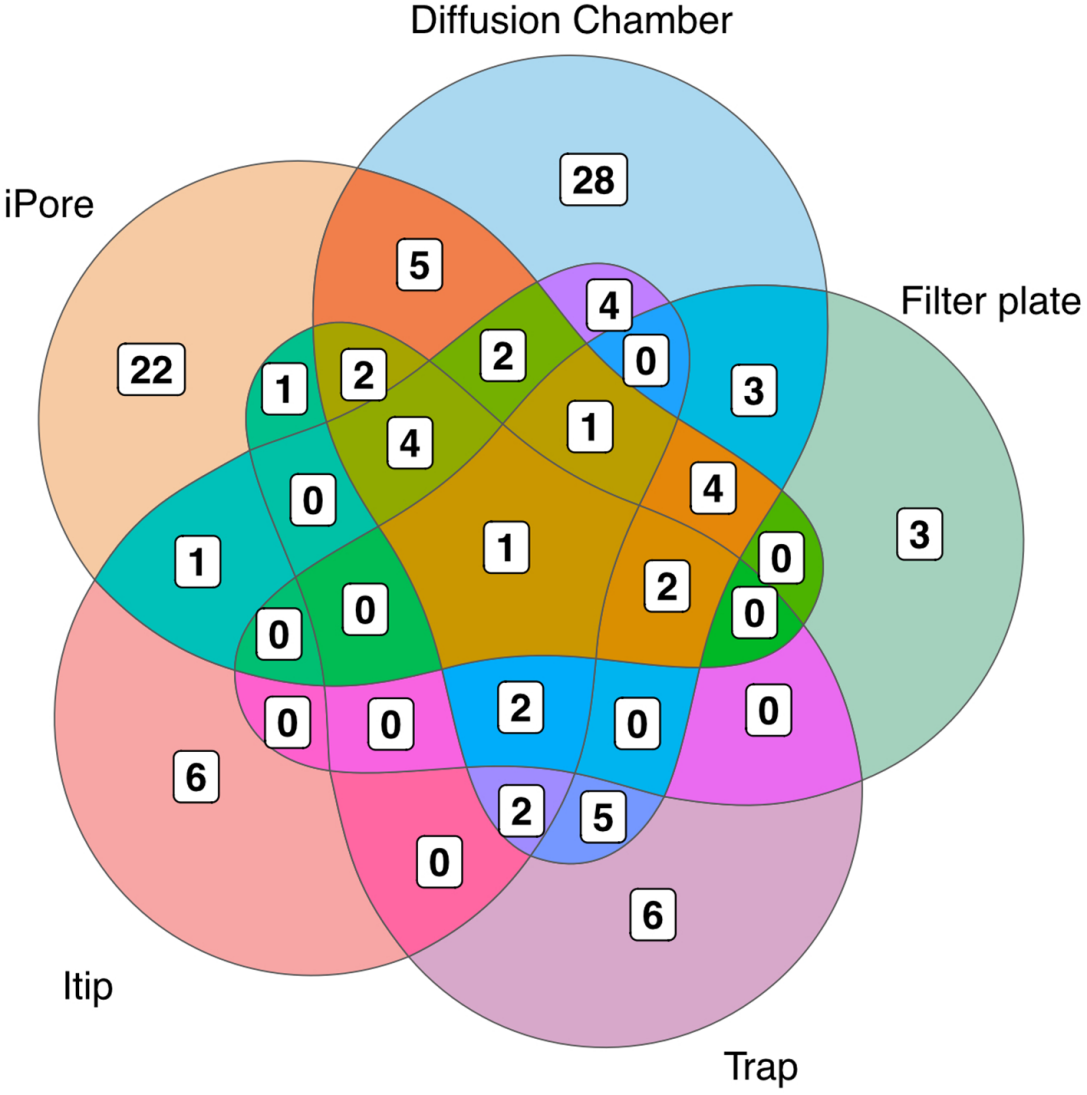
Overlap of OTUs recovered using different *in situ* methods. N indicates the total number of OTUs recovered by each method. Numbers within the diagram indicate reflect OTUs that are unique to, or shared among, *in situ* approaches, highlighting the complementary nature of these cultivation strategies.

**Figure 4:**
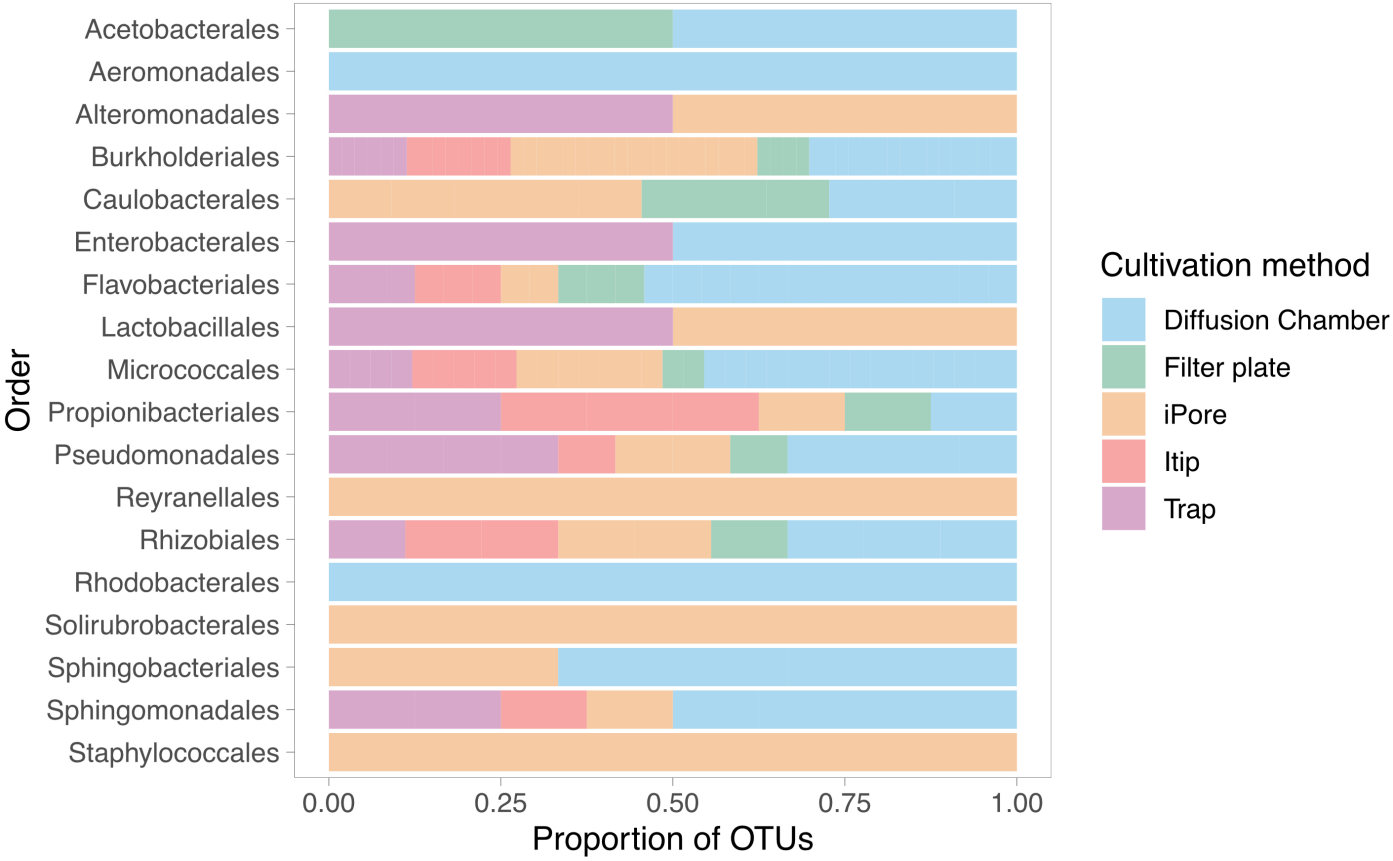
Unique *in situ* cultivation methods yield distinct taxonomic profiles among recovered isolates. Bars represent the distribution of isolates, grouped by taxonomic order of the corresponding OUT, across five *in situ* cultivation approaches. Proportions reflected the relative contribution of each method to the recovery of microbial diversity within each order.

We observed low overlap in the OTUs we cultured using each *in situ* cultivation method, with only one OTU overlapping between all methodologies (**Figure 3**). The largest number of unique OTUs were cultivated using the diffusion chamber and iPore devices. When comparing the *in situ* approaches, we observed a similar pattern as when we compare standard and *in situ* approaches as a whole, in that some orders were only successfully cultivated by some approaches (**Figure 4**). In particular, use of the iPore was necessary to culture three orders: Reyranalles, Solirubrobacterales, and Staphylococcales. Use of the diffusion chamber was necessary to culture two orders: Rhodobacterales and Aeromonadales.

### Anoxic subculture of *in situ* inoculum is required to isolate additional diversity

**Figure 5:**
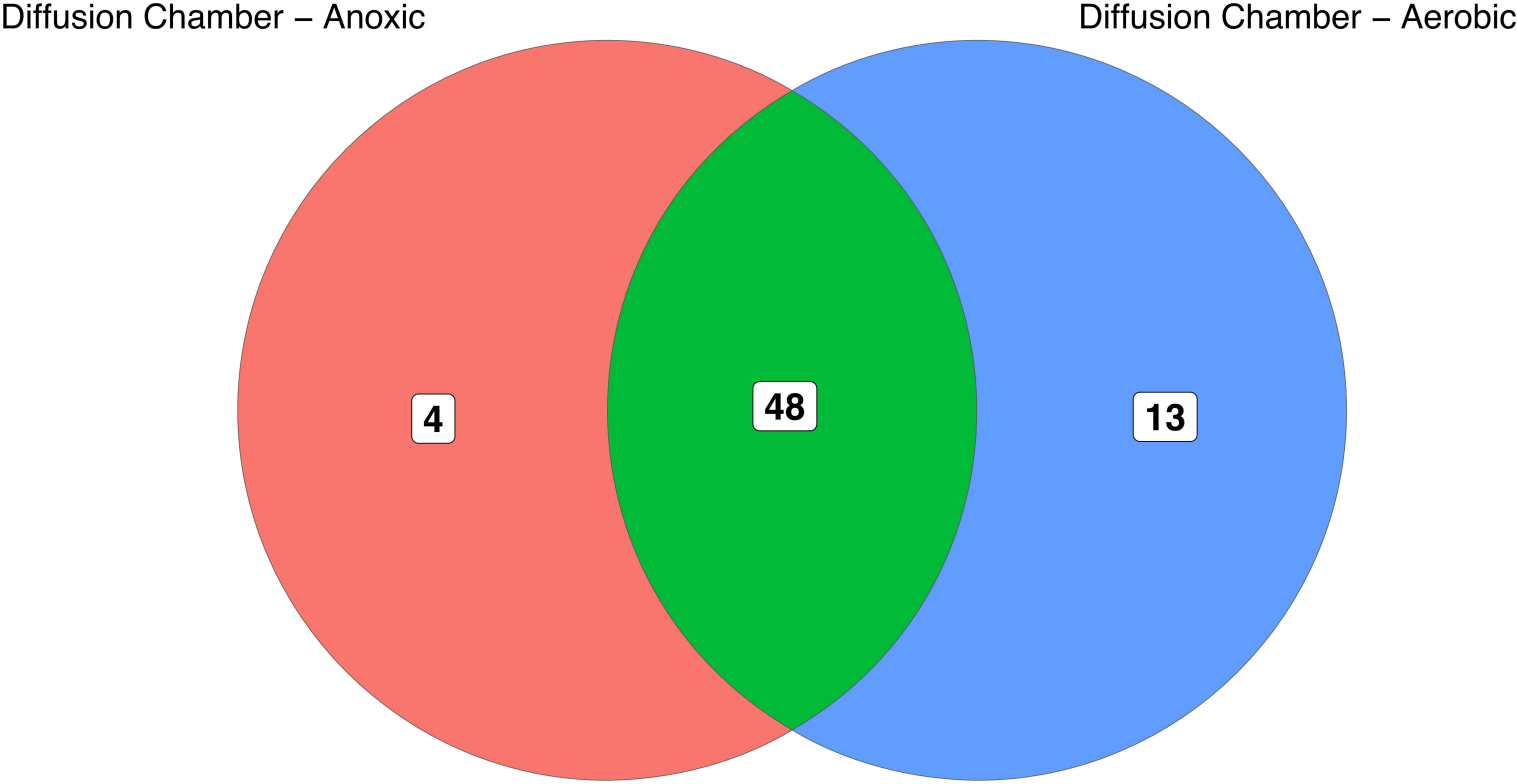
Overlap of OTUs initially recovered from diffusion chambers and subsequently subcultured under aerobic or anoxic conditions. Numbers in the diagram indicate OTUs that are unique to, or shared between, the subculturing approaches. Notably, anoxic subcultivation was required to recover seven OTUs that initially grew in diffusion chambers but could not be maintained under aerobic conditions.

We tested whether or not anoxic subculture of the colonies initially grown in the diffusion chambers increased the diversity of our culture collection. The majority of isolates initially cultured in the diffusion chamber were successfully subcultured in aerobic conditions, and 13 OTUs were cultivated using both anoxic and aerobic conditions. However, 4 OTUs required anoxic subculture conditions to be successfully isolated (**Figure 5**).

### Organisms show cultivation preference which is partially informed by phylogeny

**Figure 6:**
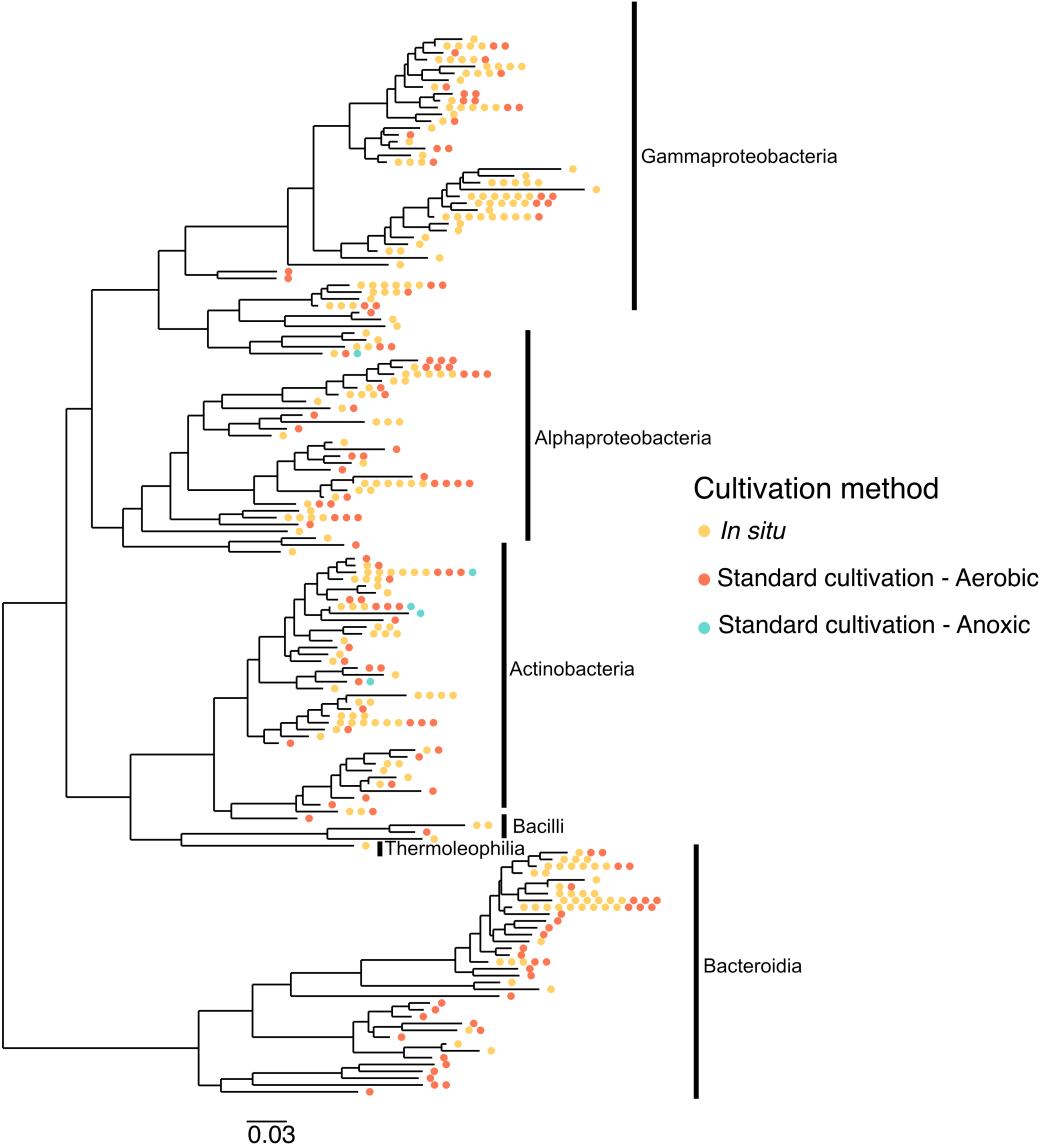
Different cultivation methodologies provide access to unique branches of the phylogenetic tree. The tree was constructed based on 16S rRNA gene sequences from isolates recovered across different cultivation strategies. Isolates span the Gammaproteobacteria, Alphaproteobacteria, Actinobacteria, Bacilli, Thermoleophilia, and Bacteroidia classes. Colored dots represent isolates obtained via *in situ* cultivation (yellow), standard aerobic cultivation (red), and standard anoxic cultivation (blue), highlighting how different methodologies access distinct regions of phylogenetic diversity.

Taxonomy played a role in which OTUs were successfully cultivated by each method. In particular, OTUs belonging to Thermoleophilia, Reyranalles, Solirubobacteriales and Staphylococcales were only cultivated using the iPore, while Streptomycetales, Xanthomonadales, Chitinophagales, and Cytophagales were only cultivated using standard aerobic approaches (**Table 3**). However, all three broad approaches recovered a relatively even spread of OTUs across the phylogenetic tree (**Figure 6**).

**Table 3:** Cultivation preference of different taxonomic groups.

Group	Most effective method for isolation
Firmicutes	Standard aerobic, iPore, trap
Thermoleophilia	*In situ*: iPore
Bacillli	Standard aerobic, *In situ*: iPore, trap
Alteromonadales	*In situ*: iPore, trap
Reyranellales	*In situ*: iPore
Solirubrobacteriales	*In situ*: iPore
Staphylococcales	*In situ*: iPore
Streptomycetales	Standard aerobic cultivation
Xanthomonadales	Standard aerobic cultivation
Chitinophagales	Standard aerobic cultivation
Cytophagales	Standard aerobic cultivation

### Cultivation methods vary in isolation novelty and efficiency

The majority of isolates recovered had a 16S rRNA gene sequence that matched between 97–100% to the closest reference sequence in the SILVA database (**Table 4**). Using standard aerobic cultivation, we isolated four OTUs with less than 96% sequence identity to their closest match in the SILVA database, with the lowest-identity isolate sharing 93% sequence similarity with a Flavobacterium species. Using standard anoxic cultivation, we recovered one isolate that shared 91.5% sequence identity with its nearest match, which belonged to the family Microbacteriaceae in the phylum Actinobacteriota. The two most novel organisms were isolated using the iPore (89%) and diffusion chamber (89%) methods (**Table 4**). The iPore isolate, which shared 89% sequence identity, was most closely related to an uncultured organism in the family Solirubrobacteraceae (phylum Actinobacteriota). Similarly, the diffusion chamber isolate with 89% sequence identity was most closely related to a member of the family Oxalobacteraceae. Incubating diffusion chamber contents under anoxic conditions also resulted in one isolate with <96% rRNA gene sequence identity to its closest SILVA match, specifically showing 95% similarity to a Mucilaginibacter species. Additionally, one isolate from the filter plate method was identified as relatively novel, sharing 95.8% rRNA gene sequence identity with a member of the family Acetobacteraceae.

**Table 4:** Number of OTUs captured using different cultivation methods, categorized by their sequence identity to the closest reference in the SILVA database. Percent identity is based on the highest similarity match found within the database. Sequences with <96% identity are considered potentially novel at the genus level, while those with <95% identity may indicate novelty at the family level or higher. Some OTUs were recovered by multiple cultivation approaches.

% identity to closest match in SILVA database	OTUs	OTUs	OTUs	OTUs	OTUs	OTUs	OTUs	OTUs
	SC - aer	SC - an	DC - aer	DC - an	T	FP	It	Ip
100	4	1	6	1	2	2	0	2
99	53	1	38	7	11	8	10	20
98	36	1	29	10	13	6	11	10
97	21	0	10	1	3	0	2	9
96	13	1	3	0	0	0	0	2
95	2	0	1	0	0	1	0	0
<95	2	1	1	1	0	0	0	2
Total OTUs captured by method	131	5	88	20	29	17	23	45

Percent identity is based on the highest similarity match found within the database. Sequences with <96% identity are considered potentially novel at the genus level, while those with <95% identity may indicate novelty at the family level or higher. Some OTUs were recovered by multiple cultivation approaches.

To analyze how methods compared to each other, we calculated isolation power to determine how many of the total available OTUs each method could capture and divided by the total number of isolates to get an isolation efficiency (**Table 5**). Standard cultivation had the largest isolation power (capturing 85% of all OTUs found in study), while the diffusion chamber had the second largest isolation power. All other isolation methods captured <30% of the OTUs detected in the study. However, when considering how this translates into diversity (isolation efficiency) the iPore had the highest isolation efficiency—even though it only captured 29% of the OTUs, it was more efficient than any other method.

**Table 5:** Comparison of cultivation methods based on isolation power, isolation efficiency, and most divergent OTU recovered.

Method	Most divergent OTU (% identity to closest SILVA match) total available	Isolation power	Isolation efficiency
Standard cultivation	93.35	0.85	0.0027
Standard cultivation (anoxic)	91.52	0.03	0.0016
Diffusion Chamber	89.46	0.57	0.0017
Diffusion Chamber (anoxic)	94.97	0.13	0.0025
Trap	97.31	0.19	0.0023
I-tip	97.03	0.15	0.0019
Filter plate	95.88	0.11	0.001
iPore	89.42	0.29	0.0028

The most divergent OTU for each method is defined as the isolate with the lowest percent identity to its closest match in the SILVA database. Isolation power is calculated as the proportion of unique OTUs recovered by each method relative to the total number of OTUs recovered across all methods. Isolation efficiency represents the fraction of unique OTUs captured per total number of isolates obtained using each method.

## Discussion

One of the major issues constraining the advancement of microbiology is the fact that many microbial species across the tree of life continue to evade cultivation. Therefore, it is important to establish best practices and develop methods that can aid in accessing the broad diversity of microbiota. We compared several *in situ* approaches with traditional aerobic and anoxic techniques to test their efficacy in cultivating microbes from lake sediment in the high Arctic. We found that 1) both *in situ* and standard approaches yielded diverse collections of microbial isolates, 2) microbial taxa exhibited cultivation preferences, with certain taxa exclusively or preferentially cultured by particular methods, and 3) no single method was sufficient to capture the full microbial diversity of the sample, emphasizing the need for a multi-method approach.

We isolated 1,109 colonies which clustered *de novo* into 155 OTUs based on 97% homology of rRNA gene sequences. Our dataset was dominated by the phylum Proteobacteria, though we also isolated members of Actinobacteria, Bacteroidota and Firmicutes. This is consistent with other culture collections from Arctic environments (Møller et al., 2013; Pearce et al., 2003; Steven et al., 2007). Within the Proteobacteria phylum, we cultivated representatives of Alpha-, Beta-, and Gammaproteobacteria, but the majority of isolates were Betaproteobacteria, which have been found to dominate freshwater systems such as our lake (Kirchman, 2002). At the order level, we encountered a majority of isolates from the orders Burkholderiales, Flavobacteriales, and Micrococcales. Interestingly, a previous study of Arctic snow encountered only one Burkholdariales isolate, in contrast with our study (Møller et al., 2013). Flavobacteriales are within the Cytophaga-Flavobacterium cluster and can be commonly found in Arctic environments, including sea ice (Staley and Gosink, 1999), cold marine surface waters (Wells and Deming, 2003), and ice-covered freshwater lakes (Møller et al., 2013). These organisms may play important roles as heterotrophs in aquatic and sea-ice environments. We also encountered a number of Pseudomonadales and Sphingomonadales, which have been detected in Arctic environments using culture-dependent methods (Steven et al., 2007). Altogether, the composition of our culture collection was largely consistent with previous work.

Interestingly, we found minimal overlap of isolates obtained by *in situ*, anoxic, and aerobic approaches: only 3 of 155 OTUs were common among all three broad methodologies (standard aerobic, anoxic, and *in situ*). We expected to find minimal overlap between anoxic conditions and other cultivation methods due to the nature of anoxic enrichments. Indeed, the OTUs we cultured using anoxic approaches were all facultative or obligate anaerobes, as expected. However, the minimal overlap between standard and *in situ* cultivation was surprising. We term the tendency of representatives of some taxonomic divisions to be cultivated by any particular approach as “cultivation preference.” Cultivation preferences became more pronounced at lower taxonomic divisions, such as order. For example, four orders were exclusively cultivated using the iPore, and four were exclusively cultivated using standard aerobic approaches. The recovery of comparable numbers of OTUs using standard cultivation was also unexpected since we cultivated twice as many isolates via *in situ* methods compared to standard cultivation. Previous studies have found that *in situ* approaches result in richer culture collections as compared to standard cultivation methodologies (Bollmann et al., 2007; Jung et al., 2016), however, our *in situ* isolate collection was not significantly richer than standard cultivation. Note, however, that *in situ* isolates described here are not what grew in the *in situ* devices but those that could be successfully subcultured from them using conventional methodologies. Further investigations could include sequencing of biomass contained in *in situ* devices to determine what taxa may be lost between initial *in situ* incubation and subcultivation in a lab.

We also found degrees of cultivation preference for microbes cultured with different *in situ* methods. This was consistent with our expectations, as each device was designed to overcome a limitation or target a specific group of organisms. The trap selects for filamentous, motile, and *Actino­*type organisms (Gavrish et al., 2008). The trap device can be easily overgrown by fast-growing species, and the filter plate was designed to overcome this limitation. It functions like a trap but contains 96 individual wells to prevent overgrowth (Jung and Ahn, 2012). The Itip was initially designed to cultivate microorganisms associated with marine sponges, and therefore has a smaller area for microbial entry, compared to the large flat surface of the filter plate and other devices (Jung et al., 2014). The iPore prototype used here selects for motile and filamentous bacteria because the only way they can reach growth chambers is through growth or movement through a maze of microfluidic channels (Tandogan et al., 2014). The diffusion chamber does not rely on motility, as it is inoculated prior to *in situ* incubation. However, multiple cells are placed in the single diffusion chamber, enabling fast growing or metabolically competitive species to easily outcompete others (Freilich et al., 2011). Given the design of each device, we expected some uniqueness in each collection. However, we were surprised to observe that the overlap of organisms cultured using the five approaches was so low—with only one OTU common to all methods. This OTU shared 98% 16S rRNA gene sequence identity with the genus *Massilia*.

In terms of novelty and efficiency, different methods exhibited varying strengths. Standard aerobic cultivation recovered the highest number of OTUs overall, but *in situ* methods, particularly the iPore and diffusion chamber, captured the most novel OTUs. The most novel organisms (89% rRNA gene sequence identity to known strains) were isolated using the iPore and diffusion chamber, highlighting their utility in accessing previously uncultured taxa. When accounting for isolation effort relative to diversity recovered, the iPore demonstrated the highest efficiency, followed by standard aerobic cultivation and the diffusion chamber. These findings suggest that while traditional methods remain effective, integrating novel *in situ* techniques can significantly enhance microbial discovery.

Due to our observation of strong cultivation preferences exhibited by particular taxonomic groups among both standard and *in situ* approaches, we suggest that a variety of cultivation methodologies should be used to more thoroughly survey a microbial community and successfully culture the microbes present therein. The strong bias of individual methods likely results from a combination of inherent device properties as well as spatial microheterogeneity in microbial distribution within the environment. Future studies should explore sequencing biomass from *in situ* devices prior to subculturing to assess potential microbial losses during laboratory processing. Additionally, refining cultivation strategies based on metagenomic insights could further optimize microbial recovery. Overall, our results highlight the importance of methodological diversity in microbial cultivation efforts. Leveraging a combination of approaches can enhance the likelihood of isolating novel and ecologically relevant microorganisms, advancing our understanding of microbial life in extreme environments and beyond.

## Data Availability

The datasets presented in this study can be found in online repositories. The names of the repository/repositories and accession number(s) can be found below: NCBI GenBank, accession PX360553–PX360705.
