# Supplementary figures and images for: Diverse cultivation strategies are necessary to capture microbial diversity in High Arctic lake sediment
Source: Front Microbiomes. 2025 Sep 26;4:1619859. doi: 10.3389/frmbi.2025.1619859 (PMC12993673; doi:10.3389/frmbi.2025.1619859)

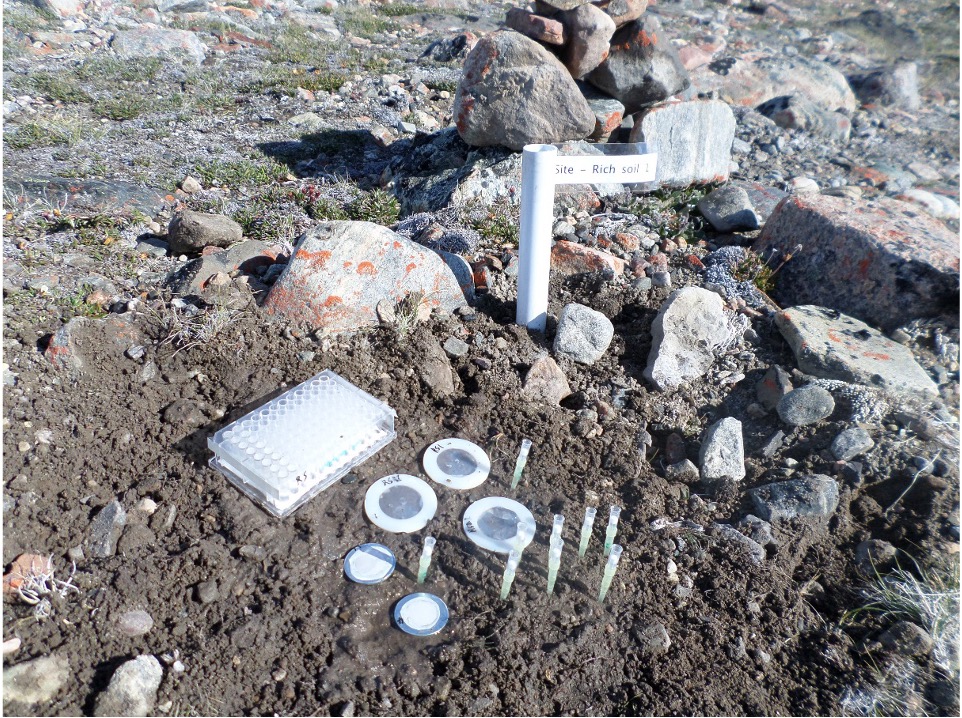

Supplement: Supplementary file 1 [file Image1.jpeg]

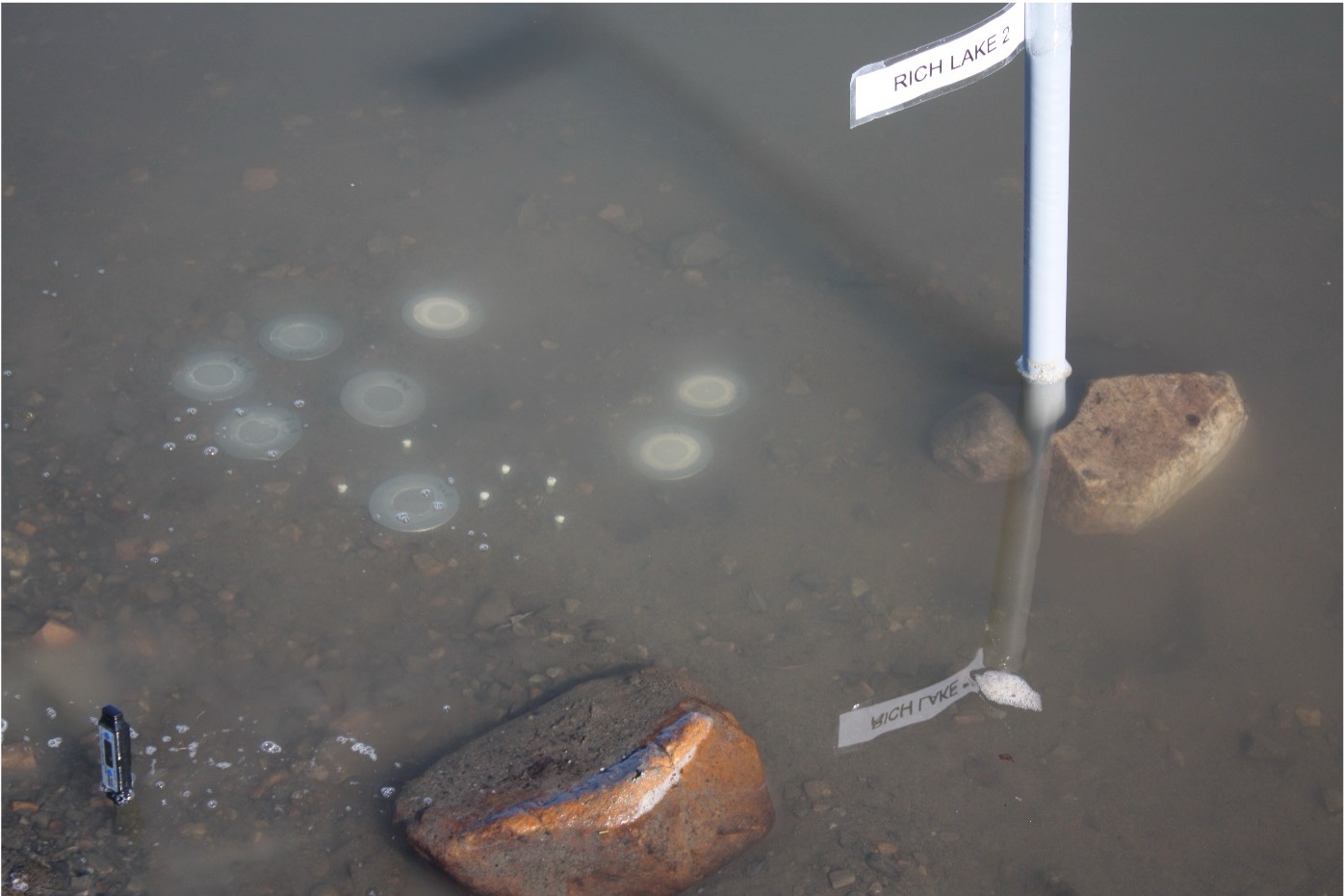

Supplement: Supplementary file 2 [file Image2.jpeg]
